# A promising strategy against SARS-CoV-2: pyrimidine inhibitors synergize with nucleoside analogues

**DOI:** 10.1038/s41392-022-00956-6

**Published:** 2022-03-15

**Authors:** Long Min, Qiu Sun

**Affiliations:** State Key Laboratory of Biotherapy, Cancer Center, West China Hospital, Sichuan University and Collaborative Innovation Center for Biotherapy, 610041 Chengdu, China

**Keywords:** Infection, Diseases

In a recent study published in *Nature*, David C. Schultz et al. demonstrated a new high-efficient therapy to fight against SARS-CoV-2, that is, combining pyrimidine biosynthetic inhibitors with antiviral nucleoside analogues.^1^

By February 10, 2022, SARS-CoV-2 has infected more than 400 million people, including more than 5.79 million deaths. Patients infected with SARS-CoV-2 usually develop mild to severe symptoms, and several patients lead to severe clinical outcomes. Although some vaccines or drugs have entered clinical practice, there is still a lack of effective antiviral therapy against the constantly changing virus.

SARS-CoV-2 is a family of single-stranded positive-sense RNA viruses. It replicates RNA by using RNA-dependent RNA polymerase (RdRp). Nucleoside analogues can interfere with this step by incorporating into the growing viral RNA chain through RdRp, then the RNA replication process will be forced to terminate or mutate, ultimately inhibiting viral replication.^2^ Nucleoside analogues have now become a large class of approved drugs that act directly as antivirals. Due to the conservative structures of RdRp in different viruses, some of the nucleoside analogues are believed to be used to inhibit SARS-CoV-2.

The respiratory epithelial cell is the main cellular target of SARS-CoV-2 in vivo. The authors used the human respiratory epithelial cell line Calu-3 to screen the small molecule compound library that included approved drugs, drugs in clinical trials, and drugs with antiviral activity with known targets.^3^ After the comprehensive evaluation of the potency and toxicity of each compound, 122 compounds of which about 13% belong to nucleoside analogues, including remdesivir and molnupiravir, which are approved to use in the treatment of SARS-CoV-2 had screened out.^4,5^ To determine the breadth of antiviral activities of these nucleoside analogues, they tested a group of cell lines which are permissive to infect with SARS-CoV-2. It was found that different nucleoside analogues showed their cell-type-specific antiviral activities. For example, tuberculin showed antiviral activities in Calu-3, Caco-2 and Huh7.5, but was toxic in A549-Ace2 and Vero cells. On the contrary, thioguanine and 6-mercaptopurine were active in Calu-3 and A549-Ace2 cells, but not active in Caco-2 or Vero cells. Among them, remdesivir and molnupiravir exhibited the highest antiviral activities. Since remdesivir is an adenosine analogue and molupiravir is a cytosine analogue, it is speculated that the combination of remdesivir and molnupiravir could show antiviral synergy, however, further studies prove that it was just an additive effect.

Nucleoside analogues can act as synthetic analogues in the replication of DNA or RNA; in addition, a subset of nucleoside analogues also works as an anti-metabolite to consume the supply of deoxynucleotides required for DNA replication or inhibit nucleoside biosynthesis enzymes by binding to metabolic enzymes and competing with natural ligands to inhibit RNA synthesis; therefore, anti-metabolite is thought to work as a broad-spectrum antiviral strategy.

There are two pathways for nucleoside biogenesis in cells, de novo synthesis and salvage pathway. The de novo synthesis can supply sufficient energy for viral replication while the salvage pathways cannot. Based on this, the researchers screened a series of compounds that inhibit nucleoside biosynthetic enzymes and found two DHODH inhibitors in de novo pyrimidine synthesis, BAY-2402234 and Brequinar, as well as the UMPS inhibitor pyrazofurin.

**Fig. 1:**
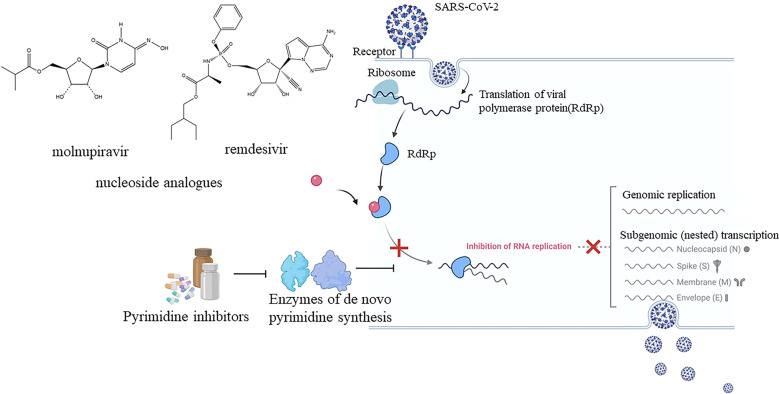
Pyrimidine biosynthesis inhibitors and antiviral nucleoside analogues synergistically inhibit SARS-CoV-2 replication in vitro and in vivo. The combining pyrimidine biosynthesis inhibitors with antiviral nucleoside analogues suggest a new clinical forward facing the emerging strains of SARS-CoV-2. Created with BioRender.com

Surprisingly, the DHODH inhibitors co-administrated with remdesivir and molnupiravir showed striking synergy. The combining therapy, DHODH inhibitors combined with remdesivir and molnupiravir, also showed positive results in diverse strains of SARS-CoV-2(alpha, beta, gamma and delta). Undoubtedly, this result brings hope to novel drug development in the treatment of SARS-CoV-2 (Fig 1).

Since late 2019, this disaster has influenced the world, even though some vaccines and drugs have been in clinical, it is urgent to develop effective drugs or therapies to combat SARS-CoV-2 due to the constant mutating that makes the virus more infective, easier to transmit, even evade vaccines and ineffective existing treatment drugs. After screening 18,000 drugs for antiviral activity by live SARS-CoV-2 infection in human respiratory epithelial cells, the David C. Schultz group found remdesivir and molnupiravir to have shown antiviral activity and selectivity against SARS-CoV-2, and further proved that the combination of remdesivir and molnupiravir with DHODH inhibitors would be effective in reducing virus replication and inflammation after SARS-CoV-2 infection. This combination therapy offers a new direction for the treatment of SARS-CoV-2 and the emerging strains. Researchers also found Paxlovid, a SARS-CoV-2 protease inhibitor, combined with molnupiravir or remdesivir against SARS-CoV-2 beta or delta strains shown an additive effect. Thus, there may be many potential combinations that need to be tested in the clinical setting, which can alter the trajectory of this terrible pandemic. It is urgent to overcome the terrible pandemic since 2019 as soon as possible, and combination therapy may be the new start point to winning the war.
